# Prediction of cesarean hysterectomy in placenta previa complicated with prior cesarean: a retrospective study

**DOI:** 10.1186/s12884-020-2790-9

**Published:** 2020-02-07

**Authors:** Bin Liu, Songqing Deng, Meifang Lin, Yimin Chen, Jian Cai, Jianbo Yang, Jinxin Zhang, Jianjian Cui, Lixia Shen, Hongning Xie, Zilian Wang

**Affiliations:** 1 Department of Obstetrics and Gynecology, The First Affiliated Hospital of Sun Yat-sen University, 58 Zhongshan Road II, Guangzhou, 510080 People’s Republic of China; 2 Department of Ultrasound, The First Affiliated Hospital of Sun Yat-sen University, Guangzhou, 510080 People’s Republic of China; 3 Department of Medical Statistics and Epidemiology, School of Public Health, Sun Yat-sen University, Guangzhou, 510080 People’s Republic of China

## Abstract

**Background:**

The prevalence of both placenta previa and cesarean are on the rise. Multiple adverse outcomes are critically increased when placenta previa is subsequent to prior cesarean. The purpose of the present study is to develop a pre-surgical method for predicting adverse outcomes in pregnancy complicated with both placenta previa and prior cesarean.

**Methods:**

Clinical data was obtained from the medical history system at the First Affiliated Hospital of Sun Yat-sen University from February 2003 to December 2016. All cases with a final diagnosis of “placenta previa/low lying placenta (ICD:O44.001-105)” and “scarred uterus complicated with pregnancy (ICD: O34.200-202)” were collected and reviewed. Hysterectomy was taken as the primary outcome; and blood loss was taken as the secondary outcome.

**Results:**

Of 219 pregnant women in the final analysis, 25 received a hysterectomy following delivery, and 48 had blood loss exceeding 1000 ml. Pre-surgical risk factors for hysterectomy are ultrasonic signs of vascular lacunae, central placenta previa, and loss of normal hypoechoic retroplacental zone. A pre-surgical predictive equation referred to as “Hysterectomy Index in Placenta Previa with Prior cesarean (HIPs)” was generated and each risk factor was weighted to create an 8-point scale. This index yielded an area under the curve of 0.972 for the prediction of hysterectomy.

**Conclusions:**

Application of the HIPs score may provide an effective pre-surgical prediction of cesarean hysterectomy in pregnant women complicated with both placenta previa and prior cesarean.

## Background

Placenta accreta is associated with multiple adverse outcomes, including massive haemorrhage, cesarean hysterectomy, and maternal mortality [1–5]. Pregnant women presenting with placenta previa and prior cesarean have a higher risk of placenta accreta [6]. In recent decades, the prevalence of placenta previa-accreta has increased [7], partly due to increasing rates of cesarean births [8].

The complications of placenta previa-accreta can be life-threatening, thus, pre-operative prediction of these adverse outcomes is of great importance [9, 10]. Several articles have provided models to predict placental invasion [11–13] in cases with placenta previa and prior cesarean. However, there currently are no models to predict adverse clinical outcomes in these patients.

Therefore, the purpose of the present study is to analyze related risk factors of adverse outcomes, including blood loss and hysterectomy, in pregnancies complicated with placenta previa and prior cesarean. A pre-surgical prediction system will be generated using risk factors that are associated with hysterectomy and blood loss.

## Materials and methods

The present study is a retrospective analysis of risk factors relating to hysterectomy and blood loss during surgery in suspected cases of placenta previa-accreta. Clinical data was obtained from the medical history system at the First Affiliated Hospital of Sun Yat-sen University from February 2003 to December 2016. All cases with a final diagnosis of “placenta previa/low lying placenta (ICD:O44.001-105)” and “scarred uterus complicated with pregnancy (ICD: O34.200-202)” were collected for primary analysis. This study was approved by the ethical committees of The First Affiliated Hospital of Sun Yat-sen University (2017–323).

Medical records were reviewed and clinical, laboratory, and ultrasonic information was collected. Clinical and laboratory information included maternal age, gestational age at delivery, time elapsed since last cesarean, number of prior cesareans, prior curettage, pregravid and prepartum BMI, neonatal birth weight, Apgar scores, and results of last hemoglobulin test prior to delivery. Ultrasonic information included four major signs related to placenta invasion (vascular lacunae, loss of normal hypoechoic retroplacental zone, retroplacental myometrial thinness, and placental thickness), as well as type of placenta previa (central, partial, marginal or low-lying) and placenta position (anterior, posterior or sidewall).

Hysterectomy was taken as the primary outcome and blood loss as the secondary outcome. The relationship between clinical, laboratory, and ultrasonic information and adverse outcomes were analyzed. Continuous and normally distributed variables were analyzed by independent sample *t* test. Categorical variables were examined with Chi-square test. Related factors with statistical significance were further scrutinized using linear logistic regression.

By applying all significant factors related to hysterectomy, we generated an evaluation system referred to as “Hysterectomy Index in Placenta Previa with Prior cesarean (HIPs)”. Each related factor was weighted to create a scale and the sensitivity and specificity were calculated for each score.

## Results

**Fig. 1:**
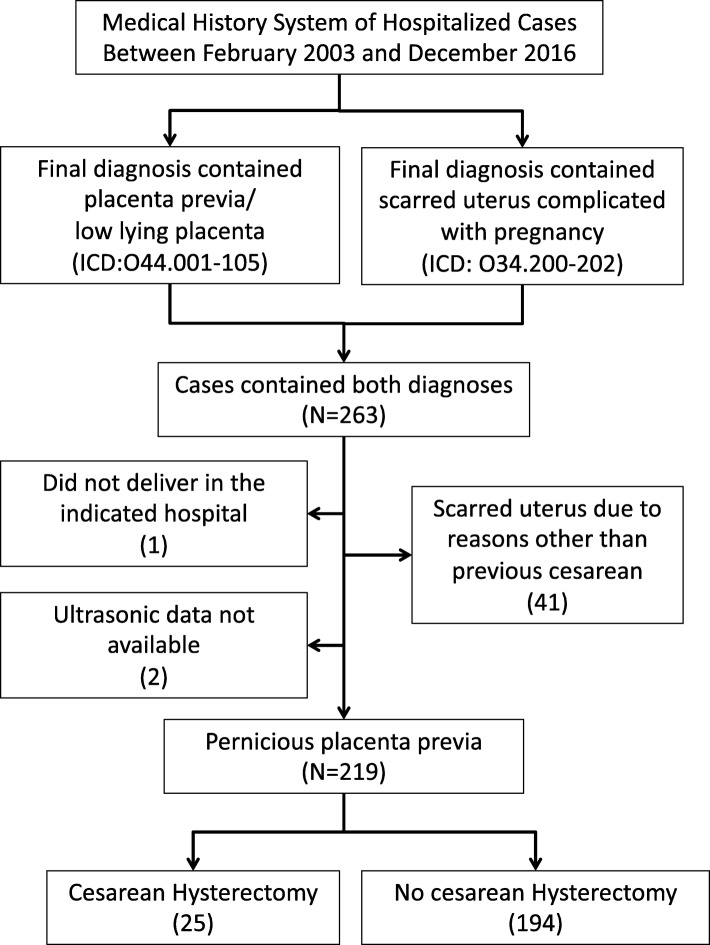
Process of clinical data collection and selection

Reviewing the database, 263 pregnant women met the inclusion criteria: diagnosis of both placenta previa and scarred uterus. Of all cases, 41 were excluded because their uterine scars were due to reasons other than previous cesarean (i.e. myomectomy), one was excluded because the patient did not deliver in our hospital, and two were excluded because ultrasonic data were not obtained due to emergency surgery. Of the remaining 219 cases in the final analysis, 25 received surgical hysterectomy (Fig. 1).

Basal maternal and neonatal information for hysterectomy and control cases are shown in Table 1. There were no statistically significant differences in age, gestational age, time since last surgery, prevalence of GDM or hypertensive disorder, bleeding history prior to delivery, pregravid and prepartum BMI, neonatal birthweight and Apgar scores. The hysterectomy group had higher incidence of two or more prior cesarean deliveries (20.0% vs 7.2%, *P* = 0.049), as well as lower hemoglobulin levels prior to delivery (103.04 ± 10.94 g/L vs 114.12 ± 15.08 g/L, *P* < 0.001). The hysterectomy group also had a higher incidence of prior curettage but did not reach statistical significance (68.0% vs 46.4%, *P* = 0.055).

**Table 1:** Basal characteristics of research population

	Hysterectomy	Control	*P* value
Number	25	194	
*Demographic characteristics and medical history*			
Age (Year)	34.08 ± 4.42	33.82 ± 4.13	0.773
Gestational age (Day)	242.44 ± 31.53	249.01 ± 34.74	0.371
Time since last surgery (Month)	55.52 ± 29.74	65.46 ± 37.64	0.205
Gestational diabetes mellitus (%)	3(12)	38(19.6)	0.585
Hypertensive disorder (%)	0(0)	7(3.6)	1.000
Prior Cesarean > = 2 times (%)	5(20.0)	14(7.2)	0.049
Prior curettage (%)	17 (68.0)	90(46.4)	0.055
Bleeding prior to delivery (%)	10(40.0)	57(29.4)	0.356
*Physical and laboratory examinations*			
Pregravid BMI (kg/m^2^)	21.83 ± 2.66	21.91 ± 2.85	0.911
Prepartum BMI (kg/m^2^)	26.09 ± 3.50	26.30 ± 2.90	0.739
Last Hb before delivery (g/L)	103.04 ± 10.94	114.12 ± 15.08	< 0.001
*Neonatal information*			
Birth weight (kg)	2.45 ± 0.62	2.78 ± 0.80	0.057
Apgar 1 min	9.23 ± 0.92	9.40 ± 1.51	0.599
Apgar 5 min	9.77 ± 0.61	9.74 ± 1.18	0.899
Apgar 10 min	9.82 ± 0.50	9.80 ± 1.14	0.931

*Hb* Hemoglobulin

*BMI* Body Mass Index

Since ultrasonic examination plays an important role in the prediction of surgery risks, we analyzed the characteristics of ultrasound images in detail. As shown in Table 2, four signs indicating placenta invasion including vascular lacunae, loss of normal hypoechoic retroplacental zone, retroplacental myometrial thinness, and placental thickness, were all more prevalent in cases with hysterectomy. In addition, the incidence of central placenta previa and anterior placenta previa were higher in patients that received hysterectomy.

**Table 2:** Ultrasonic signs related to hysterectomy and blood loss in surgery

	Hysterectomy	Control	*P* value
Number	25	194	
Vascular lacunae (%)	17(68)	16(8.2)	< 0.001
Loss of normal hypoechoic retroplacental zone (%)	22(88)	9(4.6)	< 0.001
Retroplacental myometrial thinness (%)	4(16)	5(2.6)	0.011
Placental thickness (%)	12(48)	8(4.1)	< 0.001
Central Placenta previa (%)	22(88)	56(28.9)	< 0.001
Anterior Placenta previa (%)	25(100)	102(54.8)	< 0.001

In the hysterectomy group, placenta adherence was observed in all cases with gross pathological examination, and 23 of them were further confirmed by microscopic pathology. In the 2 cases without description of placenta adherence in microscopic pathologic records, the gross manifestation of placenta adherence was obvious. One potential reason for the lack of microscopic pathology manifestation of these 2 cases may be inadequate selection of tissue from the uterus for microscopic examination.

To screen for risk factors that were associated with hysterectomy in these cases, we used logistic regression to analyze each clinical, laboratory, and ultrasonic parameter that was different in each group. As shown in Table 3, linear logistic regression demonstrated that ultrasonic indication of central placenta previa, vascular lacunae, and loss of normal hypoechoic retroplacental zone were associated with hysterectomy.

**Table 3:** Predictors of hysterectomy in pregnant women complicated with placenta previa and prior cesarean

	Regression coefficient	OR(95%CI)	*P* value
Vascular lacunae	2.041	7.701 (1.445, 41.051)	0.017
Central placenta previa	2.436	11.429 (1.395,93.618)	0.023
Loss of normal hypoechoic retroplacental zone	3.473	32.246 (5.886, 176.642)	< 0.001

Input variables include: number of cesarean> 1, prior curettage, last Hb before delivery, ultrasonic sign of vascular lacunae, loss of normal hypoechoic retroplacental zone, retroplacental myometrial thinness, placental thickness, central placenta previa, and anterior placenta previa

**Fig. 2:**
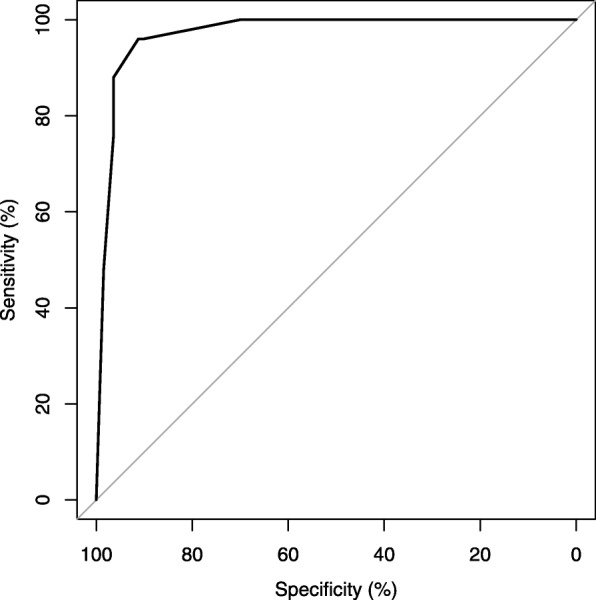
Receiver operator curves of Hysterectomy Index in Placenta Previa with Prior cesarean (HIPs). The AUC of HIPs on prediction of hysterectomy in indicative cases was 0.972(95% CI: 0.949–0.994)

Next, we generated an 8-point scale to predict the probability of hysterectomy in pernicious placenta previa cases, termed the “Hysterectomy Index in Placenta Previa with Prior cesarean (HIPs)” score (Table 4). The probability of hysterectomy for each HIPs score is shown in Table 5, with the greatest area under the receiver operating characteristic curve of HIPs at 0.972 (Fig. 2).

**Table 4:** Hysterectomy Index in Placenta Previa with Prior cesarean (HIPs)

	Score
Vascular lacunae	2
Central placenta previa	2.5
Loss of normal hypoechoic retroplacental zone	3.5

**Table 5:** Probability of hysterectomy, and sensitivity, specificity, positive and negative predictive values of HIPs scores

HIPs Score	N	Hysterectomy (%)	Probability of Hysterectomy (%)	Sensitivity (%)	Specificity (%)	PPV (%)	NPV (%)
> = 2	3	0(0)	1.867	100	68.6	29.1	100
> = 2.5	40	1(2.5)	3.093	100	70.1	30.0	100
> = 3.5	2	0(0)	8.243	96	90.2	55.8	99.4
> = 4.5	12	2(16.7)	20.184	96	91.2	58.5	99.4
> = 5.5	3	3(100)	41.585	88	96.4	75.9	98.4
> = 6	11	7(63.6)	54.430	76	96.4	73.1	96.9
=8	15	12(80.0)	90.445	48	98.5	80.0	93.6

*PPV* positive predictive value

*NPV* negative predictive value

In the present study, there was no loss of life, therefore risk factors associated with maternal mortality was not studied. However, blood loss during surgery is an important risk factor of maternal mortality, also related to the decision of hysterectomy. Therefore, we studied risk factors that related to massive blood loss (more than 1000 ml during surgery). We found that ultrasonic indication of loss of normal hypoechoic retroplacental zone, placenta thickness, and central placenta previa were related to blood loss during surgery (Table 6).

**Table 6:** Risk factors associated with massive blood loss in pregnant women complicated with placenta previa and prior cesarean

	> = 1000 mL	< 1000 mL	*P* Value	Adjusted OR (95%CI)	Adjusted *P* value
*N*	48	171			
Age (Years)	34.10 ± 4.00	33.78 ± 4.20	0.638		
Gestational age (Days)	249.8 ± 22.9	247.8 ± 36.9	0.726		
Time since last surgery (Months)	58.1 ± 33.3	66.1 ± 37.8	0.188		
Gestational diabetes mellitus (%)	11(22.9)	30(17.5)	0.407		
Hypertensive disorder (%)	0	7(4.1)	0.352		
Prior Cesarean > = 2 times (%)	10(20.8)	9(5.3)	0.002		
Prior curettage (%)	29(60.4)	78(45.6)	0.075		
Bleeding prior to delivery (%)	19(39.6)	48(28.1)	0.156		
Pregravid BMI (kg/m^2^)	22.20 ± 2.70	21.80 ± 2.86	0.403		
Prepartum BMI (kg/m^2^)	26.42 ± 3.13	26.24 ± 2.92	0.711		
Last Hb before delivery (g/L)	106.60 ± 13.17	114.61 ± 15.13	0.001		
Vascular lacunae	24(50.0)	9(5.3)	< 0.001		
Loss of normal hypoechoic retroplacental zone	29(60.4)	2(1.2)	< 0.001	115.933 (11.913,1128.228)	< 0.001
Retroplacental myometrial thinness	6(12.5)	3(1.8)	0.004		
Placental thickness	19(39.6)	1(0.6)	< 0.001	77.542 (4.394,1368.455)	0.003
Central Placenta previa	36(75.0)	42(24.6)	< 0.001	3.563 (1.287,9.861)	< 0.001
Anterior Placenta position	39(81.3)	88(54.0)	0.001		

Input parameters in logistic regression were: Prior Cesarean > = 2 times, Last Hb before delivery, vascular lacunae, loss of normal hypoechoic retroplacental zone, placental thickness, central placenta previa, anterior placenta position

## Discussion

The main challenge to clinical obstetricians on management of placenta previa-accreta is that more than half of the cases were not diagnosed prior to cesarean [14, 15]. Low pre-operative diagnosis rates can lead to increased blood loss, cesarean hysterectomy, and maternal mortality. In the present study, we analyzed related factors of cesarean hysterectomy and blood loss in suspected cases of placenta previa-accreta, and developed a predictive system termed “Hysterectomy Index in Placenta Previa with Prior cesarean (HIPs)”.

The application of HIPs can pre-operatively predict risk of cesarean hysterectomy in suspected cases, with a receiver operator curve of 0.972. Three parameters, including ultrasonic image of central placenta previa, vascular lacunae, and loss of normal hypoechoic retroplacental zone, were screened to form the HIPs point system with a total score of 8. For example, a pregnant woman with ultrasonic finding of central placenta previa and loss of normal hypoechoic retroplacental zone would receive a total score of 6, and the predicted incidence of hysterectomy would be 54.430%.

By using the HIPs score, cases with high risk of hysterectomy may be identified prior to operation, allowing additional time for full pre-surgical preparation, including ureteral stent insertion [16], verifying sufficient blood bank supplies, and arrangements for multidisciplinary therapy. In addition, patients with high risk of hysterectomy may be informed prior to surgery, which will reduce the risk of medical dispute.

In the HIPs system, ultrasonic evaluation of placental invasion plays a very important role. Ultrasonic signs indicating placental invasion, such as vascular lacunae and loss of normal hypoechoic retroplacental zone [12, 17], were included in the HIPs scoring system. This finding is consistent with previous reports on risk of placenta accreta [12]. In addition, loss of normal hypoechoic retroplacental zone and placenta thickness, other placental invasion signs, were related to massive blood loss in the present study. In a study by Yosuke Baba et al, ultrasound signs of lacunae was associated with allogeneic blood transfusion in cesarean section for placenta previa [18]. Jung-Won Kim et al found that ultrasonic signs of invasion was associated with massive transfusion in placenta previa cases [19]. These findings indicate that placenta accreta is the major cause of adverse outcomes in these cases.

There are several limitations of the present study. First, this is a retrospective study, so the predictive power of HIPs should be confirmed by a prospective study. Second, placental invasion was not confirmed in all cases, since only 25 patients received hysterectomy. A previous study [12] only included cases with histologic confirmation, so the information of those suspected patients was lost. In the present study, we focused on hysterectomy, a confirmed clinical outcome, rather than placental invasion in the whole cohort.

Despite these limitations, the present study developed a predictive system of cesarean hysterectomy in suspected cases of placenta previa-accreta, based on a full review of risk factors in a cohort spanning 10 years. To the best of our knowledge, this is the first analysis focused on adverse clinical results, including cesarean hysterectomy and massive blood loss in pregnant women complicated with both placenta previa and prior cesarean.

## Conclusion

In conclusion, the HIPs index may help clinical doctors identify high risk cases, so that more precise counseling and full preparation for delivery can be made to improve clinical outcomes. The predictive value of HIPs may be examined in subsequent prospective observations.

## Data Availability

Data of the present research is available by contacting the corresponding author on reasonable request.
